# Doxorubicin-induced oxidative stress: The protective effect of nicorandil on HL-1 cardiomyocytes

**DOI:** 10.1371/journal.pone.0172803

**Published:** 2017-02-28

**Authors:** Mari C. Asensio-López, Fernando Soler, Domingo Pascual-Figal, Francisco Fernández-Belda, Antonio Lax

**Affiliations:** 1 Cardiología Clínica y Experimental, Laboratorios de Investigación Biomédica, Universidad de Murcia en Campus de El Palmar, Murcia, Spain; 2 Departamento de Bioquímica y Biología Molecular A, Universidad de Murcia en Campus de Espinardo, Murcia, Spain; 3 Servicio de Cardiología, Hospital Clínico Universitario Virgen de la Arrixaca, Murcia, Spain; Boston University, UNITED STATES

## Abstract

The primary cardiotoxic action of doxorubicin when used as antitumor drug is attributed to the generation of reactive oxygen species (ROS) therefore effective cardioprotection therapies are needed. In this sense, the antianginal drug nicorandil has been shown to be effective in cardioprotection from ischemic conditions but the underlying molecular mechanism to cope with doxorubicin-induced ROS is unclear. Our *in vitro* study using the HL-1 cardiomyocyte cell line derived from mouse atria reveals that the endogenous nitric oxide (NO) production was stimulated by nicorandil and arrested by NO synthase inhibition. Moreover, while the NO synthase activity was inhibited by doxorubicin-induced ROS, the NO synthase inhibition did not affect doxorubicin-induced ROS. The inhibition of NO synthase activity by doxorubicin was totally prevented by preincubation with nicorandil. Nicorandil also concentration-dependently (10 to 100 μM) decreased doxorubicin-induced ROS and the effect was antagonized by 5-hydroxydecanoate. The inhibition profile of doxorubicin-induced ROS by nicorandil was unaltered when an *L*-arginine derivative or a protein kinase G inhibitor was present. Preincubation with pinacidil mimicked the effect of nicorandil and the protection was eliminated by glibenclamide. Quantitative colocalization of fluorescence indicated that the mitochondrion was the target organelle of nicorandil and the observed response was a decrease in the mitochondrial inner membrane potential. Interference with H^+^ movement across the mitochondrial inner membrane, leading to depolarization, also protected from doxorubicin-induced ROS. The data indicate that activation of the mitochondrial ATP-sensitive K^+^ channel by nicorandil causing mitochondrial depolarization, without participation of the NO donor activity, was responsible for inhibition of the mitochondrial NADPH oxidase that is the main contributor to ROS production in cardiomyocytes. Impairment of the cytosolic Ca^2+^ signal induced by caffeine and the increase in lipid peroxidation, both of which are indicators of doxorubicin-induced oxidative stress, were also prevented by nicorandil.

## Introduction

Low levels of reactive oxygen species (ROS) are constantly being generated and neutralized in cardiac myocytes, where some of them serve as intracellular regulators [1]. Alteration of the redox balance resulting in oxidative stress is an early pivotal event in the pathogenesis of several cardiac disorders, including heart failure [2,3]. In this regard, doxorubicin (Adriamycin^®^) is a potent and exogenous ROS generator that may eventually be involved in delayed cardiomyopathy when used in cancer chemotherapy. Doxorubicin-induced ROS overproduction occurs inside mitochondria and is mediated by the mitochondrial NADPH oxidase (mitoNOX) activity [4]. Furthermore, nitric oxide synthase (NOS) makes use of NADPH as reductant for the production of nitric oxide (NO) from *L*-arginine in the presence of molecular oxygen. Limiting levels of *L*-arginine or cofactor (6R)-5,6,7,8-tetrahydrobiopterin (BH_4_) lead to NOS uncoupling [5]. This circumstance favors the formation of superoxide instead of NO due to the impaired reduction of molecular oxygen by the heme iron within the NOS oxygenase domain [6]. Oxidative depletion of BH_4_ by ROS also results in enzyme uncoupling [7]. Additionally, NOS may produce superoxide at the carboxy-terminal reductase domain when doxorubicin is present [8]. The postulated mechanism involves one-electron reduction of doxorubicin to doxorubicin semiquinone and then reoxidation coupled to partial reduction of molecular oxygen to give superoxide, as in the case of mitoNOX.

Nicorandil (Ikorel^®^, Sigmart^®^) is a nicotinamide ester with a nitrate group that is used for the treatment of and protection from ischemic heart in animal models [9,10] and humans [11,12]. The attributed pharmacological effects were an increase in K^+^ conductance in cardiac cell membranes and NO donor activity [13]. Studies on ventricular myocytes demonstrated that the cardioprotective effect of nicorandil was caused by selective activation of the mitochondrial ATP-sensitive K^+^ channel (mitoK_ATP_), which was abolished by the channel blocker 5-hydroxydecanoate (5-HD) [14]. These results were consistent with previous data on reconstituted channels showing that mitoK_ATP_ and not its sarcolemmal counterpart was the target of K^+^ channel activators with cardioprotective action [15]. Other studies related with nicorandil reported anti-free radical properties in canine neutrophils [16], a scavenging effect on free radicals in streptozotocin-induced diabetic rats [17], protection from oxidative stress-induced apoptosis in cardiac myocytes [18], the inhibition of NOX expression and NOS uncoupling in streptozotocin-mediated diabetic rats [19], an improvement in the hemodynamic and mitochondrial dysfunction in doxorubicin-induced heart failure in rats [20] and, also in rat, the amelioration of parameters related with fatty liver induced by a high fat diet [21].

The ability of nicorandil to cope with ROS prompted us to study some aspects of the oxidative stress induced by doxorubicin and the protection mechanism involved, using atrial-derived HL-1 cardiomyocytes from adult mouse. This is a convenient cellular model since several phenotypic characteristics of differentiated cardiomyocytes are maintained when cultured *in vitro* [22]. The present study examines: (*i*) the implication of the NOS activity in the oxidative stress induced by doxorubicin, which impairs cytosolic Ca^2+^ signaling and increases lipid peroxidation, (*ii*) the role of nicorandil as source of NO production and protector from doxorubicin-induced ROS and (*iii*) whether mitoK_ATP_ activation and/or the NO donor activity are responsible for the protection afforded by nicorandil preincubation. The dependence of the protection by nicorandil on H^+^ movement across the mitochondrial inner membrane and the effect on the mitochondrial inner membrane potential (ΔΨ_m_) provided further clues concerning the underlying mechanism. New insights into doxorubicin-induced oxidative stress in cardiomyocytes are required since current interventions to lessen the incidence of cardiotoxicity after prolonged doxorubicin treatments are unsatisfactory.

## Materials and methods

### Reagents and media

Fluo-3/acetoxymethyl (Fluo-3/AM), Pluronic^®^ F-127, 2’, 7’-dichlorodihydrofluorescein diacetate (H_2_-DCFDA), 4-amino-5-methylamino-2’,7’-difluorofluorescein (DAF-FM) diacetate and JC-1 were Molecular Probes^®^ products from Life Technologies-Invitrogen. NADPH, pinacidil monohydrate, glibenclamide (glyburide) and KT5823 were purchased from Santa Cruz Biotechnology. GKT137831 was acquired from BioVision and the Pierce^®^ BCA protein assay kit from Thermo Fisher Scientific. Claycomb medium, fetal bovine serum, *L*-glutamine, penicillin–streptomycin mixture, norepinephrine bitartrate (A0937), the protease inhibitor cocktail (P8340) and all other reagents including doxorubicin, N_ω_-nitro-*L*-arginine methyl ester (*L*-NAME) hydrochloride and nicorandil were supplied by Sigma-Aldrich. Phosphate-buffered saline (PBS) was 137 mM NaCl, 2.7 mM KCl, 10.14 mM Na_2_HPO_4_ and 1.76 mM KH_2_PO_4_ adjusted to pH 7.4 with HCl. Tyrode medium consisted of 10 mM Hepes, 150 mM NaCl, 5.4 mM KCl, 1.2 MgCl_2_, 1.8 mM CaCl_2_, 10 mM glucose, 0.9 mM NaH_2_PO_4_ and 0.25% bovine serum albumin adjusted to pH 7.4 with NaOH. Balanced salt solution (BSS) was 5 mM Hepes, 150 mM NaCl, 4 mM KCl, 1 mM MgCl_2_, 2 mM CaCl_2_, 5.6 mM glucose and 0.9 mM NaH_2_PO_4_ adjusted to pH 7.2 with NaOH and the mitochondria isolation medium was 8 mM Na_2_HPO_4_, 1 mM NaH_2_PO_4_, 75 mM KCl and 250 mM sucrose adjusted to pH 7.0 with NaOH.

### Cellular model and doxorubicin

HL-1 cells were grown at 37°C and 5% CO_2_ in complete culture medium as previously described [23]. Plated cells were subcultured or harvested to carry out experiments when 70–80% confluence was reached. All the assays were conducted 12 h after removal of fetal bovine serum, to induce cells quiescence, antibiotics and norepinephrine. The cardiotoxic treatment was carried out by exposing the cell culture to 5 μM doxorubicin for short periods of time. The doxorubicin concentration was selected according to previous studies [4,23,24], reproducing the plasma peak concentration reached by standard infusion in patients [25].

### Cytosolic Ca^2+^ transient

Cells were seeded onto 35-mm glass bottom plates at ~4 × 10^4^ cells per plate and maintained at 37°C for 48 h in complete culture medium. After three wash cycles with prewarmed Tyrode medium cells were returned to the CO_2_ incubator ready to initiate the experiments. Before measurements, cells were loaded at 37°C for 30 min in the dark with 2 μM Fluo-3/AM in Tyrode medium supplemented with 0.02% Pluronic^®^ F-127 and 0.2 mM sulfinpyrazone. Then, plated cells were washed with Tyrode medium and left for 20 min at 25°C to complete the fluorescent probe de-esterification. The additions for each experiment are specified when described. Fluorescence intensity of plated cells was continuously monitored at 25°C using a Leica DM IRE II inverted microscope coupled to a TCS SP2 scanhead module. HCX PL APO 63x was the oil immersion objective, the numerical aperture was 1.32 and the confocal pinhole for detection was 140 μm, giving a confocal section of 1.1 μm. Samples were illuminated with the 488 nm argon-ion laser line and green fluorescence was detected at 504–530 nm. Time-dependent images were collected with a Leica DC300 FX digital camera using the customized software IM50 1.2. Cells in the field were repetitively scanned at 1 s intervals for a total duration of 15 min. Calibration at the end of each experiment was performed by adding 1 μM ionomycin to evaluate F_max_ and 40 mM EGTA aliquots until fluorescence no longer diminished to determine F_min_. The fluorescence signal was converted into free Ca^2+^ concentration using the Grynkiewicz equation [26]. The apparent dissociation constant for the Ca^2+^-Fluo-3 complex was 390 nM.

### NO production

The experimental procedure was based on the highly fluorescent benzotriazole derivative that is formed when non-fluorescent DAF-FM diacetate is nitrosated by NO after intracellular deacetylation [27]. Cells plated at ~4×10^4^ cells per well in 96-well microtiter plates were washed 3 times with prewarmed BSS, transferred to the CO_2_ incubator at 37°C and subjected to the indicated treatment. Before measurements, loading with 10 μM DAF-FM diacetate in BSS was performed at 37°C for 30 min, the final volume being 200 μl. Probe-loaded cells were again washed 3 times and maintained at 37°C for 30 min in BSS to allow the action of cytosolic esterases. NO production was evaluated at 37°C by adding 100 μM *L*-arginine to plated cells in BSS. Other reagents were added in preincubation before *L*-arginine when indicated. The time-dependent green fluorescence was recorded at 37°C using a Fluostar Omega microplate reader (BMG Labtech). The excitation and emission wavelengths were 495 and 515 nm, respectively. Experimental data correspond to fluorescence intensity in arbitrary units (a.u.) or a.u. per min with respect to a control in the absence of inhibitor and are expressed as percentage.

### Mitochondrial fraction

Cells in four 75-cm^2^ culture flasks were grown at 37°C for 3 days in complete culture medium to reach ~6 × 10^6^ cells per flask. Thereafter, they were trypsinized, pooled in Eppendorf tube and washed twice with PBS at 4°C. Cells resuspended in 400 μl of ice-cold mitochondria isolation medium were lysed at the ice-water temperature in a glass-teflon Dounce homogenizer by 80 strokes and the homogenate was centrifuged at 10,000 × g and 4°C for 20 min. The resulting pellet was resuspended in 400 μl of ice-cold medium containing 50 mM KH_2_PO_4_, pH 7.0, and 1 mM EGTA and then vigorously vortexed 5 times (10 s each time), maintaining the samples for 5 min intervals on an ice-water bath. This mitochondrial fraction was aliquoted and stored at -80°C for further study.

### MitoNOX activity

The enzymatic activity in the mitochondrial fraction was evaluated by chemiluminescence assay using the emitted light of lucigenin when reduced by the substrate NADPH [28]. The reaction was studied at 25°C using 96-well microtiter plates. In a final volume of 0.2 ml the initial reaction medium was 50 mM KH_2_PO_4_, pH 7.0, 1 mM EGTA, 150 mM sucrose and 200 μg protein from the mitochondrial fraction. The reaction was started by adding 25 μM lucigenin and 100 μM NADPH. The effect of specific reagents was studied when added in preincubation for 20 min before measurements. The photon emission in each well was measured over a timespan of 4 min using a Fluostar Omega microplate reader (BMG Labtech). Linear rates of luminescence rise in a.u. per min are presented as percentage with respect to a control in the absence of inhibitor. No enzyme activity was detected when NADPH was absent.

### Protein determination

The protein concentration in the mitochondrial fraction was evaluated with the bicinchoninic acid assay kit from Pierce^®^ and bovine serum albumin as standard protein.

### ROS measurement

Doxorubicin-induced ROS was monitored following the intracellular appearance of the fluorescent probe 2’,7’-dichlorofluorescein (DCF). Cells plated in 96-well microtiter plates at a density of ~4× 10^4^ cells per well were used. After washing 3 times with prewarmed Tyrode medium, the cells were transferred to the CO_2_ incubator where the experimental protocols were applied. Before measurements, loading with 10 μM H_2_-DCFDA in Tyrode medium (200 μl per well) was carried out at 37°C for 30 min in the dark and excess probe was removed by washing before being incubated at 25°C for 20 min. Plated cells in fresh Tyrode medium at 37°C were exposed to 5 μM doxorubicin and the time-dependent fluorescence was monitored. In some experiments, plated cells were preincubated with a certain reagent before doxorubicin addition. A Fluostar Omega microplate reader (BMG Labtech) with excitation at 485 nm and emission at 530 nm was used. Rates of DCF accumulation are expressed in a.u. per min with respect to a control in the absence of drug. Absorbance and/or autofluorescence of the drug were subtracted using cells in the presence of 5 μM doxorubicin as described previously [24].

### Evolution of ΔΨ_m_

Cells in a 96-well microtiter plate were grown at 37°C for 2 days in complete culture medium to reach ~8 × 10^4^ cells per well. Then, they were washed with prewarmed PBS and loaded at 37°C for 15 min with 5 μg/ml JC-1. After two wash cycles with PBS, cells in Tyrode medium were subjected to the corresponding treatment. In some experiments, carbonyl cyanide *m*-chlorophenylhydrazone (CCCP) was used to collapse ΔΨ_m_. The time-dependent JC-1 fluorescence was recorded using a Fluostar Omega microplate reader (BMG Labtech). The fluorescent probe was excited at 490 nm and the emission was alternately read at 530 nm and 590 nm. Data are expressed as red/green fluorescence ratio after correction for doxorubicin autofluorescence and/or energy transfer to JC-1 [4].

### Confocal images and fluorescence colocalization

Subconfluent cultures in 35-mm glass bottom plates were loaded at 37°C in Claycomb medium with 10 μM H_2_-DCFDA and then exposed to 5 μM doxorubicin for 30 min. Cells on the stage of the above described Leica equipment were examined by laser scanning confocal microscopy. The oil immersion objective was HCX PL APO 63x and the confocal section thickness was 1.1 μm. The DCF associated to doxorubicin-induced ROS and doxorubicin were excited with the 488 nm argon-ion laser line and the emitted fluorescence was detected at 500–530 nm for DCF and 620–700 nm for doxorubicin. Nicorandil and 5-HD were added in preincubation when described. Quantitative colocalization of DCF and doxorubicin was carried out by calculating the Manders overlap coefficient [4]. To this end, high magnification images of 1024 × 1024 pixels were collected and cytoplasmic regions of interest in individual cells were selected. The images were deconvoluted to improve the signal to noise ratio using the software Huygens Essential (http://svi.nl/HuygensEssential). Processing and analysis were carried out with the software ImageJ (http://imagej.nih.gov/ij/) using the Intensity Correlation Analysis (ICA) Plugin (http://wwwfacilities.uhnresearch.ca/wcif/software/Plugins/ICA.html).

### Lipid peroxidation

Malondialdehyde (MDA), the major end product of lipid peroxidation, can be quantified by reaction with thiobarbituric acid due to the formation of a red adduct [29]. After the corresponding treatment, subconfluent cultures in 6-well plates containing ~6 × 10^4^ cells per well were washed with cold PBS, scraped and lysed by homogenization in 0.1 ml of ice-cold 1.15% KCl. Cell lysate aliquots of 0.1 ml were added to a medium containing 0.2 ml SDS at 8.1% (w/v), 1.5 ml acetic acid at 20% (v/v) adjusted to pH 3.5 and 1.5 ml thiobarbituric acid at 0.8% (v/v). The mixture was brought to a final volume of 4 ml with purified water and heated at 95°C for 2 h. After cooling to room temperature each sample was mixed with 5 ml *n*-butanol and pyridine (15:1 v/v), shaken vigorously and centrifuged at 14,500×g for 10 min at room temperature. The absorbance of the resulting supernatant was measured at 532 nm with an Infinite^®^ 200 PRO spectrophotometer (Tecan Group). MDA quantitation was carried out by calibration plot using 1,1,3,3-tetraethoxypropane in the 0.25 to 4 μM range as standard.

### Data presentation

Histogram bars and data points are mean values of at least five independent assays and standard deviations are indicated by error bars. Enzyme activity data are expressed as percentage with respect to a control in the absence of inhibitor. Confocal images of fluorescence are representative of randomly selected fields and were reproduced using the Corel Photo-Paint X7 software from CorelDRAW^®^. Cytosolic Ca^2+^ transients are average of repeated experiments using more than one cell culture. Apparent rate constants for Ca^2+^ transient decay correspond to mean values ± SEM. Differences between mean values were analyzed by the Student’s *t* test using SigmaPlot 11.0 from Systat Software; *p* < 0.05 was considered significant.

## Results

### NOS and mitoNOX activities

**Fig 1:**
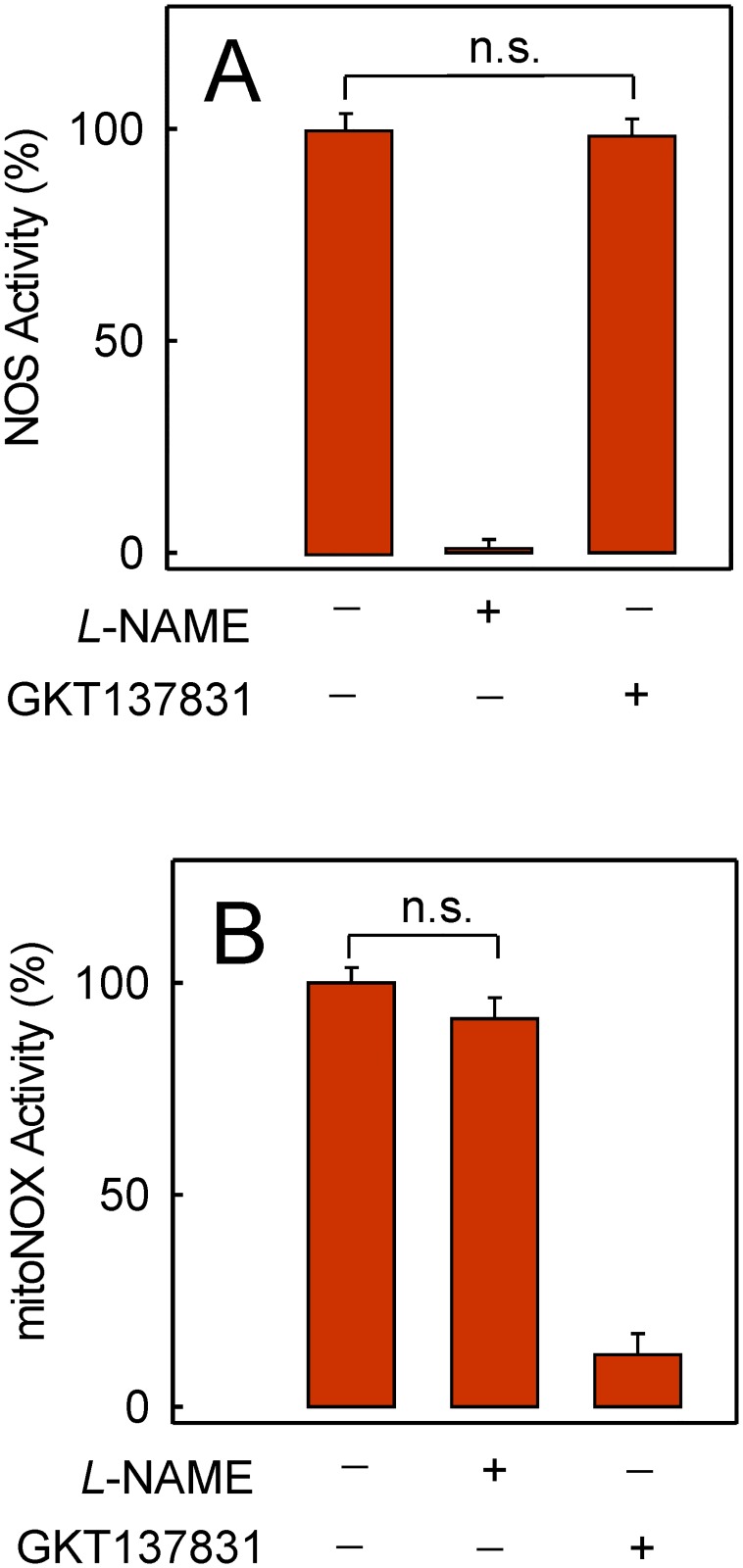
Sensitivity of NOS and mitoNOX activities to specific inhibition. (A) NOS activity was measured in plated cells loaded with 10 μM DAF-FM diacetate. After loading and de-esterification (30 + 30 min), the reaction at 37°C was started by adding 100 μM *L*-arginine. When indicated, 100 μM *L*-NAME or 1 μM GKT137831 was added in preincubation for 20 min before measurements. (B) mitoNOX activity was measured at 25°C in samples from the mitochondrial fraction using the lucigenin assay as detailed in the experimental section. When indicated, 100 μM *L*-NAME or 1 μM GKT137831 was added 20 min before the reaction was started. n.s. means no significant difference (*p* > 0.05).

NOS and NOX are ROS-related enzymes, endowed with a heme group within an electron transport chain for molecular oxygen reduction, that can be selectively inhibited. As shown, the NOS activity in plated cells was 99% inhibited by 100 μM *L*-NAME but was insensitive to 1 μM GKT137831 (Fig 1A). In contrast, the mitoNOX activity measured in the isolated mitochondrial fraction was insensitive to 100 μM *L*-NAME but was 90% inhibited in the presence of 1 μM GKT137831 (Fig 1B).

**Fig 2:**
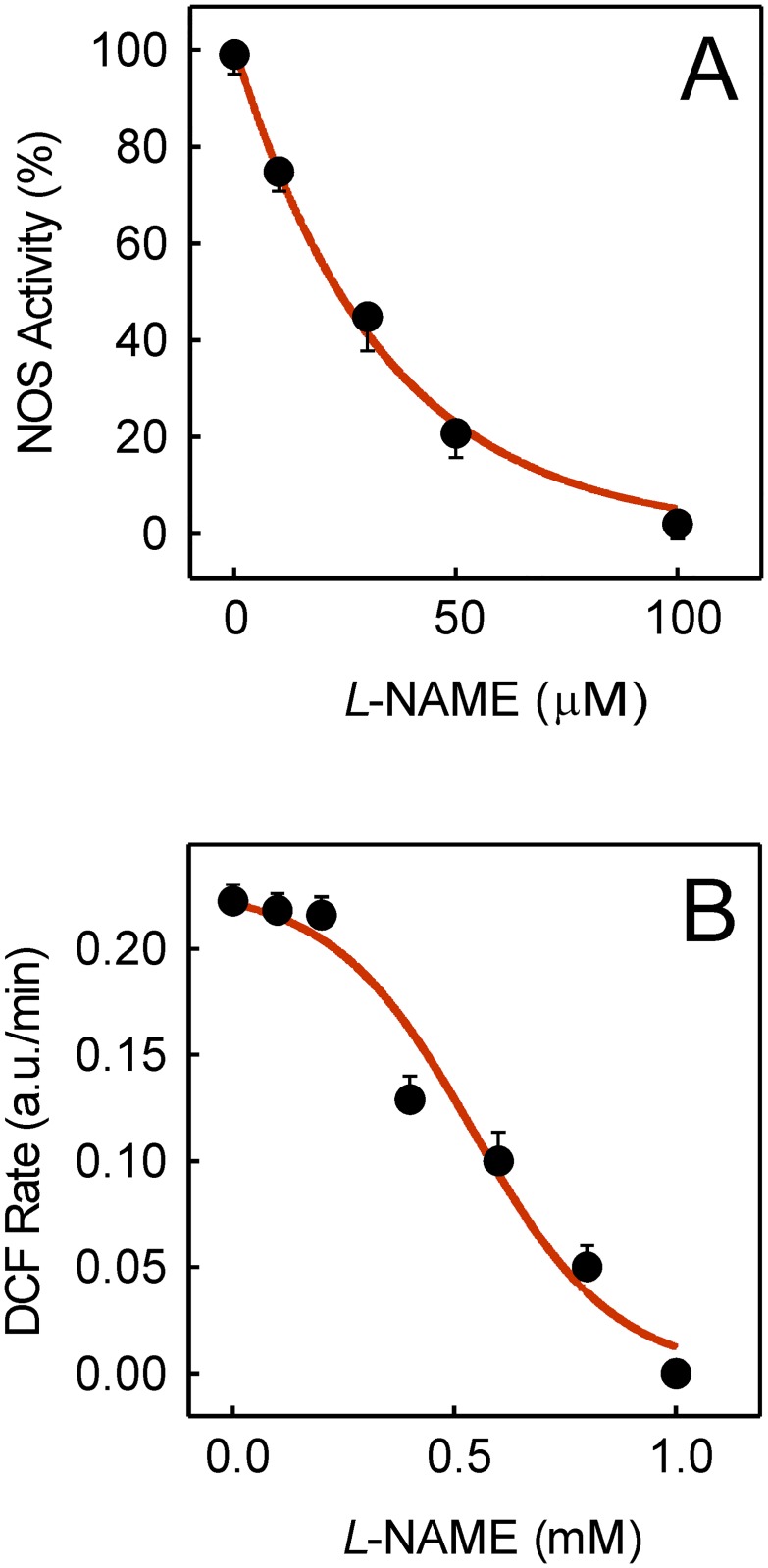
Effect of *L*-NAME on NOS inhibition and doxorubicin-induced ROS. (A) Rates of NOS activity were measured in plated cells at 37°C loaded with the DAF-FM probe. When indicated, μM *L*-NAME was added in preincubation for 2 h before the reaction was started. (B) Rates of ROS production were measured at 37°C in Tyrode medium when plated cells were loaded with 10 μM H_2_-DCFDA and 5 μM doxorubicin was added to initiate the process. Preincubation with mM *L*-NAME for 30 min was carried out before doxorubicin addition when indicated.

MitoNOX inhibition by GKT137831 prevented doxorubicin-induced ROS in a concentration-dependent manner when added in preincubation [4]. However, the relationship between NOS inhibition and the protection of doxorubicin-induced ROS in HL-1 cells has not yet been established. Our data show that NOS activity was progressively inhibited as the *L*-NAME concentration added in preincubation was raised. Practically, full inhibition was attained in the presence of 100 μM *L*-NAME (Fig 2A). However, when plated cells were preincubated with 100 μM *L*-NAME before exposure to 5 μM doxorubicin, *L*-NAME did not prevent doxorubicin-induced ROS (Fig 2B). In fact, doxorubicin-induced ROS protection by *L*-NAME became evident at concentrations higher than those required to inhibit NOS activity. Full protection was reached at 1 mM.

### NO and nicorandil

**Fig 3:**
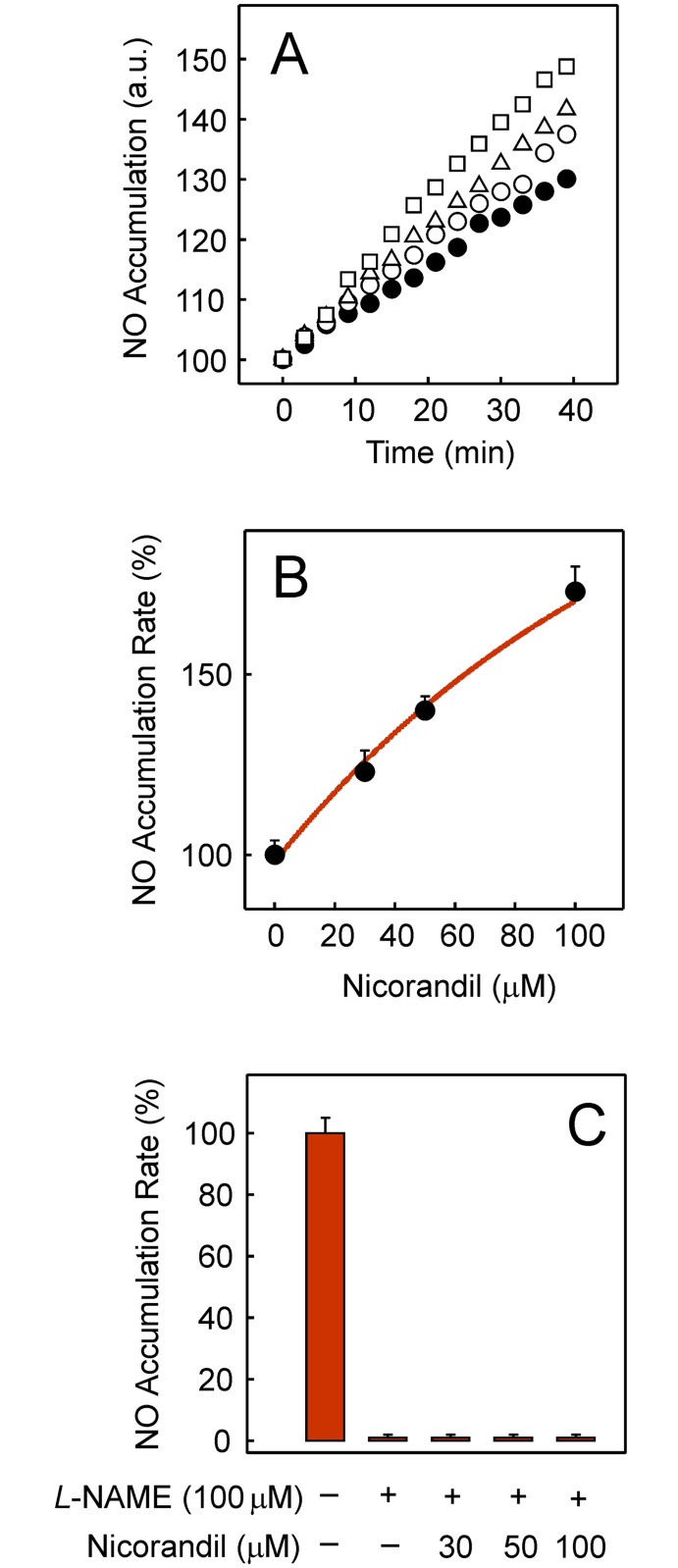
Effect of nicorandil on NO production. (A) Time-course of the intracellular NO appearance when plated cells at 37°C were preincubated for 90 min in the absence (●) or presence of 30 (○), 50 (Δ) or 100 μM nicorandil (□) before the reaction was started. (B) Dependence of the NO production rate on nicorandil concentration when added in preincubation for 90 min. Data are expressed with respect to the rate measured in the absence of nicorandil (100%). (C) NO production rate before and after preincubation with 100 μM *L*-NAME for 30 min and effect when the preincubation with *L*-NAME was prolonged for 90 min in the presence of nicorandil before measurements.

Intracellular NO production can be monitored by a linear fluorescence increase over time when HL-1 cells were loaded with the DAF-FM probe and supplemented with 100 μM *L*-arginine (Fig 3A). Moreover, the NO accumulation rate was activated when nicorandil was added in preincubation before the addition of *L*-arginine. The dependence of the NO production rate on nicorandil concentration clearly showed how the NO production was stimulated by nicorandil (Fig 3B). When data were plotted on a relative scale, the full rate of endogenous activity was observed in the absence of *L*-NAME and complete inhibition was patent when cells were preincubated with 100 μM *L*-NAME for 2 h before the reaction was started (Fig 3C). Interestingly, the NO accumulation rate was negligible when different nicorandil concentrations were added 30 min after 100 μM *L*-NAME and preincubation was prolonged for 90 min.

### NOS activity and doxorubicin

**Fig 4:**
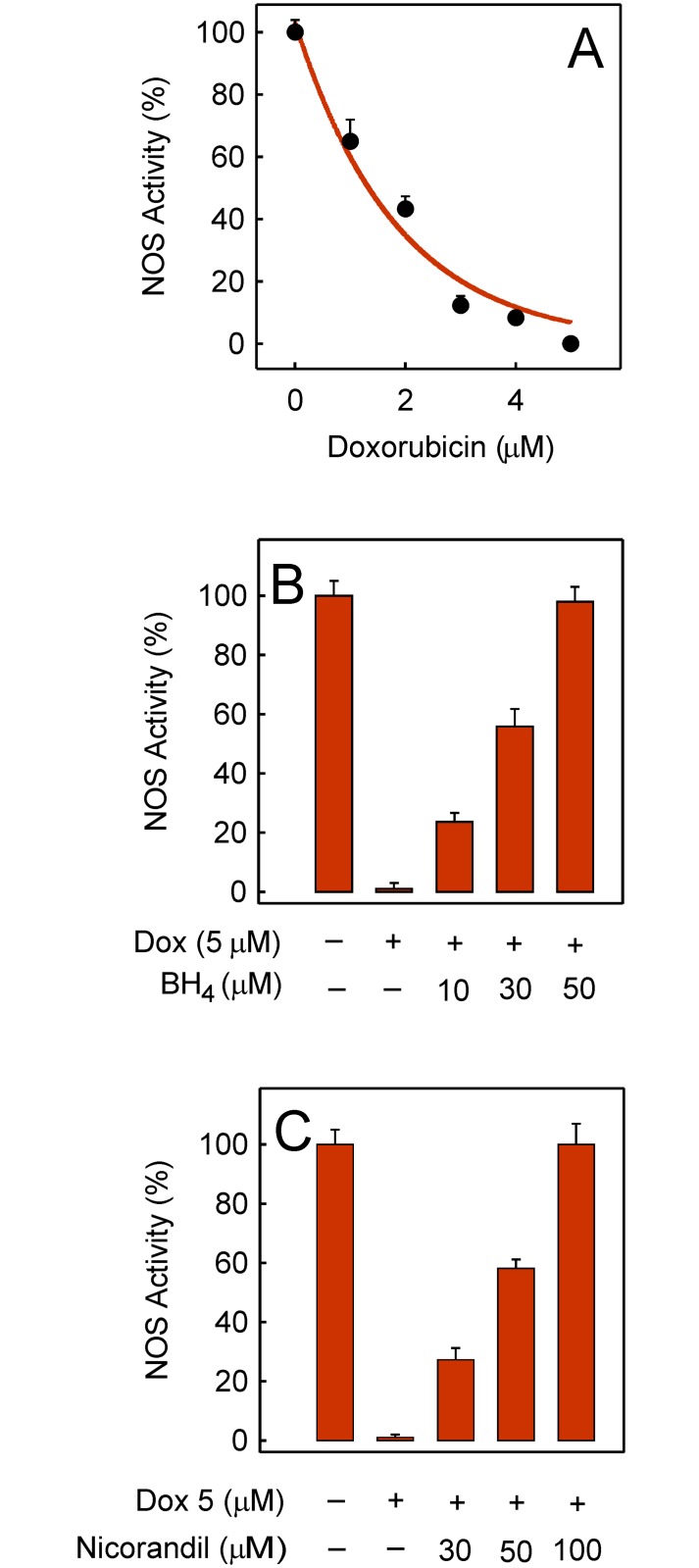
Inhibition of the NOS activity by doxorubicin and protection by BH_4_ or nicorandil. Rates of NO accumulation were measured at 37°C after exposing plated cells to doxorubicin for 1 h. (A) Effect of the doxorubicin concentration when added 1 h before the reaction was started by *L*-arginine. (B) Effect of the BH_4_ concentration when added 30 min before 5 μM doxorubicin (Dox) and the treatment was prolonged for 1 h before *L*-arginine addition. (C) Effect of the nicorandil concentration when added in preincubation for 30 min before 1 h treatment with 5 μM doxorubicin (Dox) and then the reaction was started.

The NOS activity was sensitive to doxorubicin as shown when plated cells were exposed to doxorubicin for 1 h. The inhibition of NOS activity by doxorubicin-induced ROS was concentration-dependent and full effect was developed when the doxorubicin concentration reached ~5 μM (Fig 4A). It can also be seen that BH_4_ added in preincubation for 30 min before the 5 μM doxorubicin treatment protected the NOS activity in a concentration-dependent way (Fig 4B). In similar experiments, nicorandil instead of BH_4_ was added in preincubation for 30 min before the 5 μM doxorubicin treatment. In this case too, the NOS activity was protected by nicorandil in a concentration-dependent manner (Fig 4C).

### ROS and nicorandil

**Fig 5:**
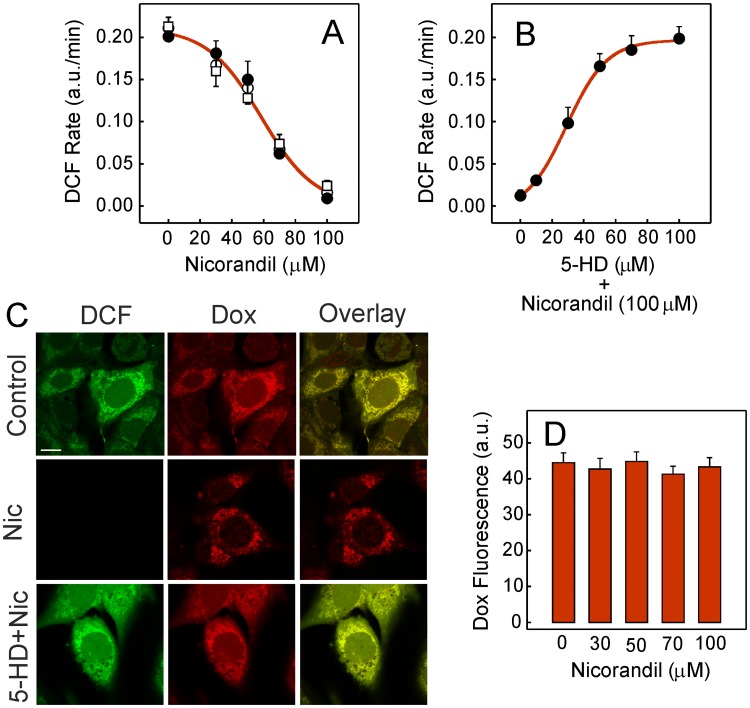
Characteristics of the nicorandil-mediated protection against doxorubicin-induced ROS. Rates of ROS production were measured at 37°C in Tyrode medium following the DCF accumulation when the process was started by adding 5 μM doxorubicin. (A) Dependence on nicorandil concentration when added in preincubation for 30 min before measurements (●) or when the preincubation with nicorandil included 100 μM *L*-NAME (○) or 1 μM KT5823 (□). (B) Dependence on 5-HD when added in preincubation with 100 μM nicorandil for 30 min before measurements (●). (C) Fluorescence confocal images from live cells once loaded with 10 μM H_2_-DCFDA and exposed to 5 μM doxorubicin for 30 min. Control images of DCF production (green) induced by doxorubicin (red) (*upper row*). Effect of 100 μM nicorandil (Nic) when added in preincubation for 30 min before exposure to doxorubicin (*middle row*). Effect of 100 μM 5-HD when added in preincubation with 100 μM nicorandil for 30 min before the 30 min treatment with doxorubicin (*lower row*). Superimposed images from the corresponding left and middle panels are shown on the right. (D) Red fluorescence was measured by confocal microscopy when plated cells were exposed to 5 μM doxorubicin for 30 min before or after 30 min preincubation with different nicorandil concentrations. Plotted data are average of mean fluorescence ± SEM in 20 μm^2^ cytoplasmic regions (*n* = 20) from randomly selected cell fields after background subtraction.

Plated HL-1 cells gave rise to a linear rate of ROS production as soon as 5 μM doxorubicin was added. However, when the cell culture was preincubated for 30 min with nicorandil before doxorubicin addition, the rate of ROS production declined following a sigmoidal dependence as the nicorandil concentration was raised (Fig 5A). Maximal inhibition required ~100 μM nicorandil. The inhibition profile of doxorubicin-induced ROS was exactly the same when 100 μM *L*-NAME or 1 μM KT5823 was added 30 min before the 30 min preincubation with different nicorandil concentrations. Additional experiments were carried out by including the putative selective mitoK_ATP_ inhibitor 5-HD before nicorandil in the preincubation step. As shown, the rate of ROS production was minimal when the 30 min preincubation was performed in the presence of 100 μM nicorandil and the absence of 5-HD (Fig 5B). However, when the 5-HD concentration added in preincubation with 100 μM nicorandil was raised before exposure to doxorubicin, the rate of ROS production gradually increased. The nicorandil effect on doxorubicin-induced ROS was also studied by fluorescence confocal microscopy. In these assays, plated cells that were first loaded with 10 μM H_2_-DCFDA and then exposed to 5 μM doxorubicin for 30 min displayed both green and red fluorescence due to the ROS production induced by doxorubicin (Fig 5C, *upper row*). Colocalization of the fluorescent signals was inferred from the yellow color of the overlaid image, which provided a Manders overlap coefficient of 0.98 ± 0.01. When 100 μM nicorandil was added in the 30 min preincubation before the 30 min treatment with 5 μM doxorubicin the green fluorescence was no longer observed and the overlaid image only showed the presence of doxorubicin (*middle row*). Likewise, when the preincubation step for 30 min included 100 μM 5-HD plus 100 μM nicorandil, DCF accumulation induced by doxorubicin was reestablished and the overlaid image showed colocalization of green and red fluorescence (*lower row*). The potential effect of nicorandil on the intracellular accumulation of doxorubicin was also assessed as a control by measuring red fluorescence. Accordingly, plated cells were exposed to 5 μM doxorubicin during a time period of 30 min before or after 30 min preincubation with nicorandil. The mean fluorescence value of doxorubicin remained unaltered whether or not HL-1 cells were previously treated with a given nicorandil concentration (Fig 5D). Therefore, no effect on doxorubicin metabolism or uptake into cardiomyocytes can be attributed to the presence of nicorandil.

**Fig 6:**
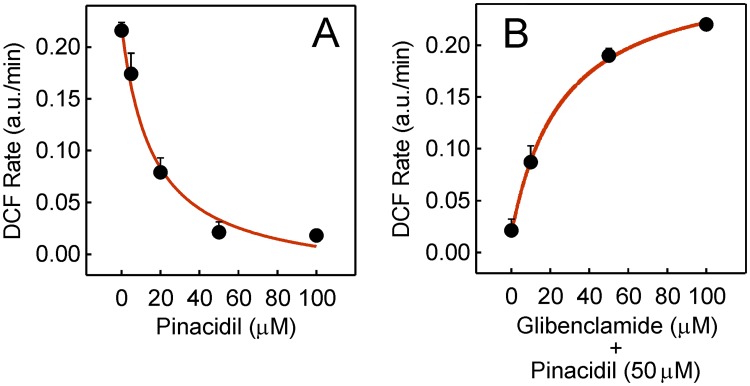
Sensitivity of doxorubicin-induced ROS to other agents. (A) Dependence of the rate of ROS production on pinacidil concentration when added in preincubation for 30 min before the addition of 5 μM doxorubicin. (B) Dependence on glibenclamide concentration when added in preincubation with 50 μM pinacidil for 30 min before doxorubicin.

The protective effect of nicorandil on mitochondrial ROS production that was abolished by the presence of 5-HD prompted us to explore the response of mitoK_ATP_ to other effectors. Thus, the rate of ROS production induced by 5 μM doxorubicin was progressively inhibited as the concentration of the non-selective K_ATP_ activator pinacidil added in preincubation for 30 min was raised (Fig 6A). Full inhibition was attained at concentrations higher than 50 μM. When the experiment was repeated by including the general K_ATP_ inhibitor glibenclamide in preincubation with pinacidil, the protection afforded by pinacidil was abolished in a concentration-dependent fashion (Fig 6B). Full recovery of ROS production required at least 100 μM glibenclamide.

**Fig 7:**
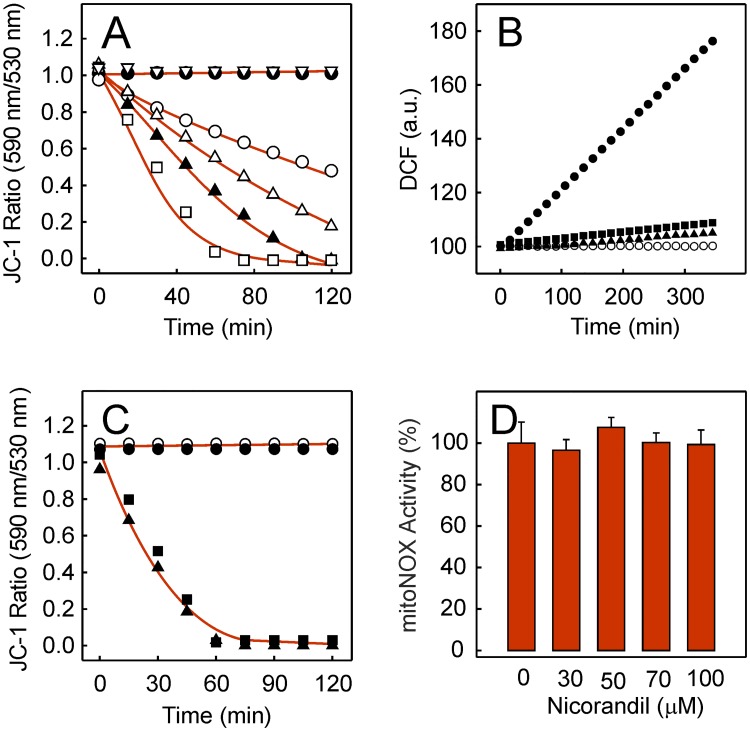
Role of ΔΨ_m_ in the mechanism of protection by nicorandil. (A) Time-course of ΔΨ_m_ when cells were left untreated (●), supplemented with 30 (○), 50 (Δ), 70 (▲) or 100 μM nicorandil (□) or supplemented with 100 μM 5-HD before the 100 μM nicorandil addition (▽). (B) Time-course of ROS accumulation in the absence (○) or presence of 5 μM doxorubicin (●) and effect of 5 μM CCCP (■) or 1 mM ZnCl_2_ (▲) when added in preincubation before doxorubicin. (C) Evolution of ΔΨ_m_ when cells were left untreated (○) or exposed to 5 μM doxorubicin (●), 5 μM CCCP (■) or 1 mM ZnCl_2_ (▲). (D) Initial rates of mitoNOX activity were measured in samples from the mitochondrial fraction before or after preincubation with a given nicorandil concentration.

Since mitoK_ATP_ opening by nicorandil would result in mitochondria depolarization, the time-dependent evolution of ΔΨ_m_ was monitored with the JC-1 probe. Live cells maintained constant levels of the red/green fluorescence ratio due to the mitochondrial accumulation of JC-1 (Fig 7A) and the nicorandil addition was followed by a time- and concentration-dependent decrease of the JC-1 fluorescence ratio. Nonetheless, cells displayed unaltered ΔΨ_m_ when 100 μM 5-HD was added 15 min before 100 μM nicorandil. Further characteristics of the protection mechanism were uncovered by studying the dependence of doxorubicin-induced ROS accumulation on H^+^ movement and the involvement of ΔΨ_m_. The time-dependent increase of DCF when 5 μM doxorubicin was added, that was not observed in the absence of doxorubicin, was taken as a positive and negative control, respectively, of induced oxidative stress (Fig 7B). Notably, when cells were preincubated with 5 μM CCCP for 10 min before the addition of doxorubicin, ROS production was virtually eliminated. There was also an absence of ROS accumulation when 1 mM ZnCl_2_ was used in the 10 min preincubation instead of CCCP. When the time-dependent effect of these agents on ΔΨ_m_ was tested, untreated cells displayed a constant ΔΨ_m_ value that was unaffected during the observed time window by the addition of 5 μM doxorubicin (Fig 7C) as already reported [4]. However, the addition of either 5 μM CCCP or 1 mM Zn^2+^ led to a time-dependent decrease of ΔΨ_m_. The effect of nicorandil on mitoNOX activity that is the source of doxorubicin-induced ROS [4] was also evaluated. The mitoNOX activity was 23.3 a.u. per min when samples from the isolated mitochondrial fraction were used. No effect on enzyme activity was observed when samples were preincubated with nicorandil before measurements (Fig 7D). This confirmed that mitoNOX activity was not directly inhibited by nicorandil.

### Protective role of nicorandil

**Fig 8:**
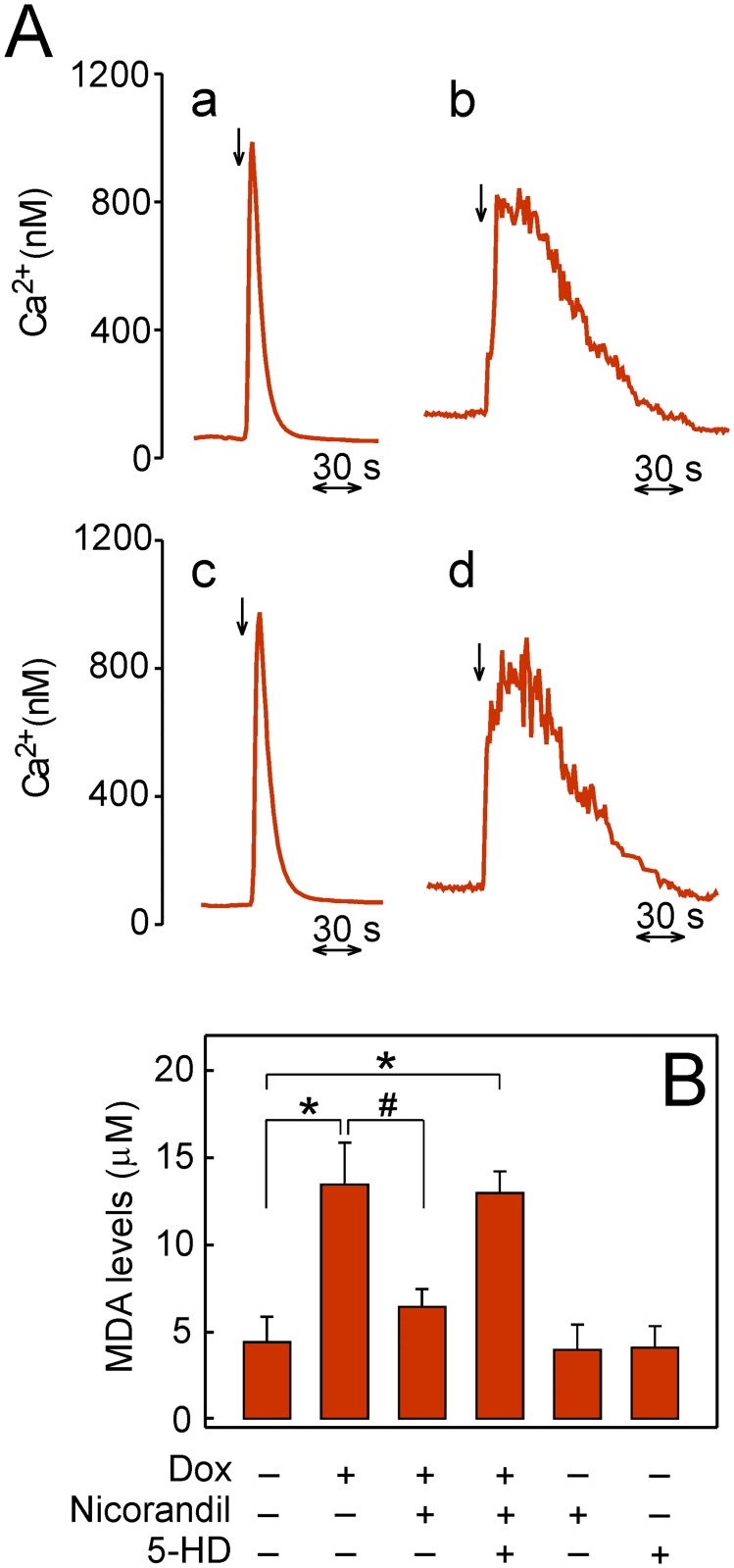
Protection of cytosolic Ca^2+^ transient and lipid peroxidation by nicorandil preincubation. (A) Plated cells were loaded with Fluo-3/AM and maintained in Tyrode medium at 37°C during the experiments. Fluorescence trace of cytosolic free Ca^2+^ before and after application of 10 mM caffeine (arrow) for the irreversible discharge of the sarcoplasmic reticulum Ca^2+^ store (*trace a*). Ca^2+^ transient induced by 10 mM caffeine after exposing plated cells to 5 μM doxorubicin for 1 h (*trace b*). Cells were preincubated with 100 μM nicorandil for 30 min and then exposed to 5 μM doxorubicin for 1 h before caffeine addition (*trace c*). Cells preincubated with 100 μM 5-HD plus 100 μM nicorandil for 30 min were then exposed to 5 μM doxorubicin for 1 h before caffeine addition (*trace d*). Traces are average of repeated experiments (*n* = 5) collected from confocal images of representative cell fields. (B) MDA content as index of lipid peroxidation when plated cells were treated or not with 5 μM doxorubicin (Dox) for 1 h, preincubated for 30 min with 100 μM nicorandil in the absence or presence of 100 μM 5-HD before doxorubicin treatment, or exposed to nicorandil or 5-HD alone. Significant difference *vs*. control (*). Significant difference between absence and presence of nicorandil (#).

Intracellular Ca^2+^ transporters involved in cardiac contractility are early targets of doxorubicin [4]. Thus, plated HL-1 cells in Tyrode medium exhibited a cytosolic free Ca^2+^ at rest of 59 ± 7 nM. The application of 10 mM caffeine (*n* = 5) triggered a Ca^2+^ peak value of 985 ± 35 nM and the rate constant for Ca^2+^ transient decay was 155 ± 18 ms^-1^ (Fig 8A, *trace a*). However, when plated cells were exposed to 5 μM doxorubicin for 1 h the caffeine-induced Ca^2+^ transient was clearly affected (*trace b*). The resting level was 125 ± 16 nM Ca^2+^, the peak amplitude reached 798 ± 29 nM Ca^2+^ and the rate constant for Ca^2+^ transient decay was 15 ± 3 ms^-1^. The Ca^2+^ transient was clearly restored when 100 μM nicorandil was added in preincubation 30 min before the 1 h treatment with 5 μM doxorubicin and then caffeine was applied (*trace c*). It is also shown that the presence of 100 μM 5-HD together with 100 μM nicorandil in the 30 min preincubation before the 1 h doxorubicin treatment recreated the deleterious effect of doxorubicin on the caffeine-induced Ca^2+^ transient (*trace d*). It was also observed that 100 μM nicorandil added in preincubation for 90 min without doxorubicin treatment had no effect on the caffeine-induced Ca^2+^ transient. In order to check the effect of doxorubicin-induced ROS on lipid peroxidation, we evaluated the MDA content before and after the treatment. Thus, a low level of lipid peroxidation was observed when HL-1 cells were cultured in the absence of doxorubicin (Fig 8B). However, when the cell culture was exposed to 5 μM doxorubicin for 1 h there was a 3-fold increase in MDA. Furthermore, preincubation with 100 μM nicorandil for 30 min before doxorubicin treatment decreased this MDA value. The protection of lipid peroxidation by nicorandil was canceled when 100 μM 5-HD was included in the preincubation medium. Additional controls showed that neither nicorandil nor 5-HD affected the basal level of MDA when added in the absence of doxorubicin.

## Discussion

*L*-NAME is a prodrug of the active inhibitor N_ω_-nitro-*L*-arginine that is currently used as a potent inhibitor of the NOS activity *in vitro* and *in vivo* [30], whereas the NOX1/NOX4 specific inhibitor GKT137831 has been shown to inhibit mitoNOX activity [4]. In this respect, functional similarities between NOS and mitoNOX include the use of NADPH as electron-donor for the catalytic activity and an electron transport chain for the reduction of molecular oxygen. The specificity of the inhibition by *L*-NAME or GKT137831 (Fig 1) throws light on the contribution of the NOS activity to the oxidative stress induced by doxorubicin. Thus, *L*-NAME was effective as NOS inhibitor at 10 to 100 μM, while doxorubicin-induced ROS was blocked by *L*-NAME in the 100 μM to 1 mM range (Fig 2). These data clearly indicated that ROS blocking by mM *L*-NAME was unrelated with the process of NOS inhibition. Nor can the protection from doxorubicin-induced ROS by mM *L*-NAME be attributed to inhibition of the mitoNOX activity since mM *L*-NAME does not affect cellular events dependent on NOX activity [31].

Endogenous NO is generated from the amino acid *L*-arginine by NOS but may also be formed from several organic nitrates used as anti-ischemic drugs in different subcellular locations and by different mechanisms [32]. Bioactivation of the organic nitrate nicorandil when incubated with isolated mitochondrial fractions was described as being dependent on NADPH through an unidentified enzymatic activity [33]. In line with these findings, our data show that intracellular NO production is stimulated by nicorandil (Fig 3A and 3B) and that the process is totally prevented by *L*-NAME (Fig 3C), an inhibitor of an NADPH-dependent enzyme. Hence, the present results point to NOS as being responsible for NO production from nicorandil in HL-1 cells.

The NOS activity requires enzyme dimerization since the electrons flow from the reductase domain of one monomer to the heme group in the oxygenase domain of the other monomer [34]. In fact, BH_4_ aids activation of heme-bound molecular oxygen in the oxygenase domains of the NOS homodimers [35] and restores dimerization and NOS activity [36]. The rate of NOS activity that gradually decreased when the doxorubicin concentration was raised revealed that NOS was a target of doxorubicin (Fig 4A). Besides, the rate of ROS production generated by partial NOS uncoupling due to doxorubicin [8] was negligible compared with doxorubicin-induced ROS mediated by mitoNOX activity. This can be inferred from the lack of effect on doxorubicin-induced ROS when *L*-NAME was raised to 100 μM to fully inhibit the NOS activity (Fig 2B). Furthermore, the observed loss of NOS activity can be attributed to oxidative depletion of BH_4_ since preincubation with exogenous BH_4_ before doxorubicin treatment restored the NO production capacity (Fig 4B). Experimental oral treatment with BH_4_ was shown to improve pathological dysfunctions in a mouse model of cardiac hypertrophy and fibrosis [37] although the benefits of BH_4_ in clinical trials have not been reproduced. Nicorandil also protected the NOS activity (Fig 4C) but the effect is presumed to be permanent since nicorandil blunted ROS generation (Fig 5A).

The doxorubicin-induced ROS in HL-1 cells that are formed by mitoNOX and inhibited by GKT137831 [4] can be fully prevented by nicorandil in a concentration-dependent manner (Fig 5A). The cardioprotective effect of nicorandil observed up to 100 μM in a cellular ischemia model was previously attributed to the selective activation of mitoK_ATP_ [14]. In this regard, the dependence of doxorubicin-induced ROS on nicorandil was not attenuated when NO production was blocked by *L*-NAME or the activation of cGMP-dependent protein kinases was blocked by KT5823. In addition, the nicorandil effect was totally neutralized by 5-HD (Fig 5B). These findings indicate that the cardioprotection afforded by nicorandil was not related with the NO donation activity. It is known that mitochondria are early targets of doxorubicin in HL-1 cells and DCF derived from doxorubicin-induced ROS is early detected inside mitochondria in this cell type [4]. Therefore, the absence of DCF fluorescence when cells were preincubated with nicorandil before doxorubicin treatment and the recovery of the DCF fluorescence when the preincubation was carried out in the presence of 5-HD plus nicorandil (Fig 5C) confirmed the location of the nicorandil effect. These data are consistent with the preferential location of nicorandil in the mitochondrial fraction when given orally to rats [38] and also with the nicorandil bioactivation observed in isolated mitochondria from rat heart [33]. Additional evidence of the nicorandil effect as activator of mitoK_ATP_ was the induced decrease of ΔΨ_m_ that was avoided by 5-HD (Fig 7A). Moreover, doxorubicin-induced ROS was prevented by preincubation with the K_ATP_ activator pinacidil and the pinacidil effect was cancelled by the antidiabetic sulfonylurea derivative and K_ATP_ inhibitor glibenclamide (Fig 6A and 6B). It should be stressed that nicorandil did not alter the intracellular doxorubicin content (Fig 5D) and direct inhibition of the mitoNOX activity by nicorandil can be discarded (Fig 7D). The cytosolic Ca^2+^ transient induced by caffeine that is sensitive to doxorubicin-induced ROS [4], and the lipid peroxidation associated to ROS overproduction that is a component of the cardiotoxic effect of doxorubicin [39,40] were both protected by preicubation with nicorandil and unprotected when 5-HD was added with nicorandil (Fig 8).

MitoK_ATP_ activation is recognized as a key component of several cardioprotection mechanisms [14,15] although the exact role played is still unclear. Several lines of evidence sustain the functional interplay between NOX and mitoK_ATP_. For instance, the increase in endothelial ROS observed at the onset of global lung ischemia was markedly blocked by pretreatment with the nonspecific NOX inhibitor diphenyleneiodonium [41] or the K_ATP_ activator cromakalim [42]. Likewise, hepatic oxidative stress with elevated NADPH activity in a hiperlipidemic mouse model was protected by mitoK_ATP_ activation [43]. Besides, ROS production induced by angiotensin II, when infused before coronary occlusion for ischemic preconditioning, was canceled by the nonspecific NOX inhibitor apocynin or the mitoK_ATP_ inhibitor 5-HD [44]. The preconditioning effect of angiotensin II was attributed to ROS generated by cytosolic NOX that was the signal for mitoK_ATP_ activation. In consequence, ΔΨ_m_ depolarization was followed by extensive release of ROS through the permeability transition pore that was the final effector [45]. Other studies on ischemic preconditioning showed that mitoK_ATP_ activation by diazoxide was the trigger for ROS generation inside the mitochondria [46]. In this case, the source of protective ROS was suggested to be site III of the electron transport chain and the protection was blocked by 5-HD. It is significant that the proposed models of cardioprotection have overlooked the role of mitoNOX as the main contributor to ROS production in cardiomyocytes.

It is documented that one-electron transfer from cytosolic NADPH to extracellular molecular oxygen catalyzed by NOX is an electrogenic process [47]. The NOX catalytic activity is voltage- and pH-dependent [48] and the coupled H^+^ efflux for charge compensation occurs through an H^+^ channel located on the catalytic subunit [49]. Our data reveal that ROS production induced by doxorubicin was dependent on H^+^ movement since it was sensitive to the protonophore CCCP and the H^+^ channel blocker Zn^2+^ (Fig 7B). Moreover, interference of the H^+^ movement by either CCCP or Zn^2+^, which provided protection from ROS, was linked to a decrease in ΔΨ_m_ (Fig 7C). On the other hand, mitoK_ATP_ regulates several mitochondrial functions, including ΔΨ_m_ [50]. Thus, the activation of mitoK_ATP_ by nicorandil will cause K^+^ entry into the mitochondria, and therefore, ΔΨ_m_ decrease (Fig 7A) as already shown for pinacidil and other K_ATP_ activators [50]. In turn, ΔΨ_m_ depolarization by nicorandil will cause inhibition of the electrogenic NOX. Indeed, mitoK_ATP_ and mitoNOX are both redox sensitive. In our experimental system, mitoNOX inhibition by GKT137831 (Fig 1B) and [4] or mitoK_ATP_ activation by nicorandil leading to a decrease in ΔΨ_m_ (Fig 7A) offered full protection against the deleterious ROS production induced by doxorubicin. In this connection, the oxidative stress induced by hypoxia-reoxygenation in endothelial cells was suggested to be blocked by nicorandil via NOX inhibition [51].

In conclusion, although nicorandil exhibits a dual pharmacological action, the cardioprotective effect was exerted by mitoK_ATP_ opening without the participation of the NO/cGMP-dependent pathway. It is suggested that mitoK_ATP_ activation by nicorandil, giving rise to mitochondrial depolarization, is responsible for mitoNOX inhibition and the subsequent lack of ROS production. Modulation of ΔΨ_m_ may be a promising therapeutic strategy for the treatment or prevention of cardiac disorders related with ROS overproduction.
